# Analysis of patient‐specific quality assurance for Elekta Unity adaptive plans using statistical process control methodology

**DOI:** 10.1002/acm2.13219

**Published:** 2021-03-23

**Authors:** Sarah Strand, Amanda Boczkowski, Blake Smith, Jeffrey E Snyder, Daniel Ellis Hyer, Sridhar Yaddanapudi, David A. P. Dunkerley, Joel St‐Aubin

**Affiliations:** ^1^ University of Iowa Hospitals and Clinics Iowa City IA USA

**Keywords:** MRI‐guided adaptive radiotherapy, Elekta Unity, patient specific quality assurance

## Abstract

The Elekta Unity MR‐linac utilizes daily magnetic resonance imaging (MRI) for online plan adaptation. In the Unity workflow, adapt to position (ATP) and adapt to shape (ATS) treatment planning options are available which represent a virtual shift or full re‐plan with contour adjustments respectively. Both techniques generate a new intensity modulated radiation therapy (IMRT) treatment plan while the patient lies on the treatment table and thus adapted plans cannot be measured prior to treatment delivery. A statistical process control methodology was used to analyze 512 patient‐specific IMRT QA measurements performed on the MR‐compatible SunNuclear ArcCheck with a gamma criterion of 3%/2 mm using global normalization and a 10% low dose threshold. The lower control limit (LCL) was determined from 68 IMRT reference plan measurements, and a one‐sided process capability ratio $\left(C_{p,l}\right)$ was used to assess the pass rates from 432 measured ATP and 80 measured ATS plans. Further analysis was performed to assess differences between SBRT or conventional fractionation pass rates and to determine whether there was any correlation between the pass rates and plan complexity. The LCL of the reference plans was determined to be a gamma pass rate of 0.958, and the $C_{p,l}$ of the measured ATP plans and measured ATS plans were determined to be 1.403 and 0.940 for ATP and ATS plans, respectively, while a $C_{p,l}$ of 0.902 and 1.383 was found for SBRT and conventional fractionations respectively. For plan complexity, no correlation was found between modulation degree and gamma pass rate, but a statistically significant correlation was observed between the beam‐averaged aperture area and gamma pass rate. All adaptive plans passed the TG‐218 guidelines, but the ATS and SBRT plans tended to have a smaller beam‐averaged aperture area with slightly lower gamma pass rates.

## 1 INTRODUCTION

MRI‐guided adaptive radiotherapy (MRIgRT) machines are revolutionizing personalized radiation therapy by allowing day‐to‐day treatment plan adaptation.^1^, ^2^, ^3^ These adaptations, based on a daily MR image, allow for more targeted treatment plans than traditional treatment planning where a single plan could be used for several weeks depending on the fractionation scheme. Under the traditional treatment planning methodology, a patient’s radiation treatment plan could be re‐planned only a small number of times if there was a noticeable change in patient contour due to weight loss or if there was thought to be a clinically relevant change in tumor size.^4^, ^5^, ^6^

For complex treatment plans, such as intensity modulated radiation therapy (IMRT) plans, patient‐specific quality assurance (QA) tests are often performed prior to patient treatment. The American Association of Physicist in Medicine (AAPM) Task Group (TG) 218 provides contemporary patient‐specific QA recommendations.^7^ Specifically, AAPM TG‐218 recommends a universal action limit set at a 90% pass rate with a 3%/2 mm gamma criteria using global normalization and a 10% dose threshold for any IMRT treatment plan prior to patient treatment.^7^ The AAPM TG‐218 guidelines are given for standard or traditional conditions where it is possible to test the patient plan on a QA phantom prior to their treatment.

With clinical care now including MRIgRT treatments, this standard QA process is no longer possible. For a MRIgRT treatment, the first (or reference) patient treatment plan is completed prior to treatment, so it is possible to run the reference plan on a QA phantom prior to treatment. However, for each treatment session, the patient will receive an MR scan during the procedure. This scan will reflect the location of the tumor and organs at risk (OAR) for that fraction, and the original reference treatment plan can be adapted to match the patient’s current position and anatomy. These adaptations allow for improved clinical care ensuring target coverage and minimizing dose to OAR uniquely for each delivered fraction.

One of the commercially available MRIgRT machines is the Elekta Unity (Crawley, UK). The Elekta Unity is comprised of a Philips Marlin 1.5 T MRI and a standing‐wave linear accelerator generating a 7 MV flattening filter free (FFF) radiation beam. In the Unity MR‐linac workflow, Adapt to Position (ATP) and Adapt to Shape (ATS) options are available to adapt the treatment plan to the daily patient position and anatomy as seen on the MRI.^8^ For an ATP re‐plan, the daily MR is rigidly registered to the planning CT, and the MLCs are adapted to the changed position creating a virtual couch shift. An ATS re‐plan is a full treatment re‐plan with contour adjustments and new segment and final fluence calculations are based on the daily MR images. ATS plans use the same Hyperion optimizer in Monaco whereas the ATP plans use a different optimizer based on a warm‐start optimization.

Because MRIgRT treatments consist of a new IMRT plan that is generated for each fraction while the patient lies on the treatment table, patient‐specific QA measurements using traditional phantoms are not possible before the treatment commences. Since the daily‐adapted treatment plans cannot be measured using current equipment before the daily‐adapted plan is delivered to the patient, an investigation into the QA pass rates of the adapted plans compared to the initial reference plan is needed to provide insight into the variations between the daily adapted plans on the Unity MR‐linac. To our knowledge, the variability or consistency of the gamma pass rates for each daily ATS or ATP plan to their reference plan has not been investigated.

For this study, statistical process control (SPC) is used to compare the results of the measured gamma pass rates of the adaptive ATP and ATS plans to the measured gamma pass rates of the original reference treatment plans. Further analysis on the plan complexity, determined by calculating the modulation degree and beam‐averaged aperture area, was performed for the reference, ATP and ATS plans to determine if there were any correlations between these metrics and the measured gamma pass rates. Historically, SPC has been used in radiotherapy studies to determine the effect of a procedure change in an experimental population, such has the dosimetric changes to a treatment plan when using alternative planning techniques.^9^, ^10^, ^11^, ^12^ Statistical process control is a method of statistical analysis that is used to evaluate the function efficiency of a reference process and monitor the variation of additional experimental processes. As such, SPC can provide a statistical expectation of how a given ATP or ATS treatment will be delivered based on the deliverability of its reference plan.

## 2 MATERIALS AND METHODS

### 2.A Elekta unity adaptive planning

During each daily‐fractionated treatment, the patients were instructed to lie on the table with proper immobilization in place. Following an MRI acquisition, a physicist and physician worked together to generate an adapted treatment plan, either ATP or ATS. ATP uses a rigid registration to align the daily MR and planning CT datasets. The CT dataset or reference plan is treated as the primary dataset, and the daily MRI is shifted to align with the primary dataset. After the registration is approved, there are four options for optimizing the treatment plan within the ATP workflow using: (a) original segments, (b) adapt segments, (c) optimize weights, and (d) optimize shapes.^8^ A plan calculated with “original segments” aligns the two datasets and uses the original MLC pattern on the new patient position. This is only appropriate for instances where there are minimal shifts. A plan calculated with “adapt segments” moves the MLC positions using a segment aperture morphing (SAM) algorithm^13^ and projects the fluence to the new patient position through the adapted MLC positions. A plan calculated with “optimize weights” uses the SAM algorithm to modify the MLC positions and optimizes the beam weights to best match the original DVH parameters under the new patient position conditions. Finally, a plan calculated with “optimize shapes” is a re‐optimization of both the MLC shapes and weights using a warm‐start gradient descent optimization algorithm that aims to match the reference plan DVH. Machine deliverability constraints are imposed in each optimization loop of the warm‐start optimizer.

Plans calculated under the ATS workflow experience a full deformable image registration (DIR) of the CT to the MR. In many cases, the DIR adapted contours need to be further adjusted to match the daily anatomy as seen on the MRI. Following contour generation and approval, ATS uses the full Monaco Hyperion optimizer with the DVH constraints set during the reference plan creation. These constraints can be adjusted in real‐time during the re‐optimization process as needed. As a result, an entirely new re‐optimized plan is generated from both ATP and ATS procedures with the exception of ATP original segments, albeit through different means. Both ATP and ATS adapted planning workflows are performed while the patient lies on the treatment table within the MR‐linac bore. As a result, it is not possible to perform QA of the adapted plan before the patient is treated. The policy at our institution was to have the patient‐specific QA completed prior to the subject’s next treatment fraction.

#### 2.A.1 IMRT plan creation and QA measurements

Patients were treated on the Unity MR‐linac at a single institution. The patients were first imaged using a Siemens (Erlangen, Germany) Biograph 40 positron emission tomography (PET)/computed tomography (CT) scanner for dose calculations, and a Siemens Magnetom Tim Trio 3 T magnetic resonance (MR) scanner. The MR and CT data sets are registered in the Monaco treatment planning software, where an initial reference plan was created.

**Fig. 1:**
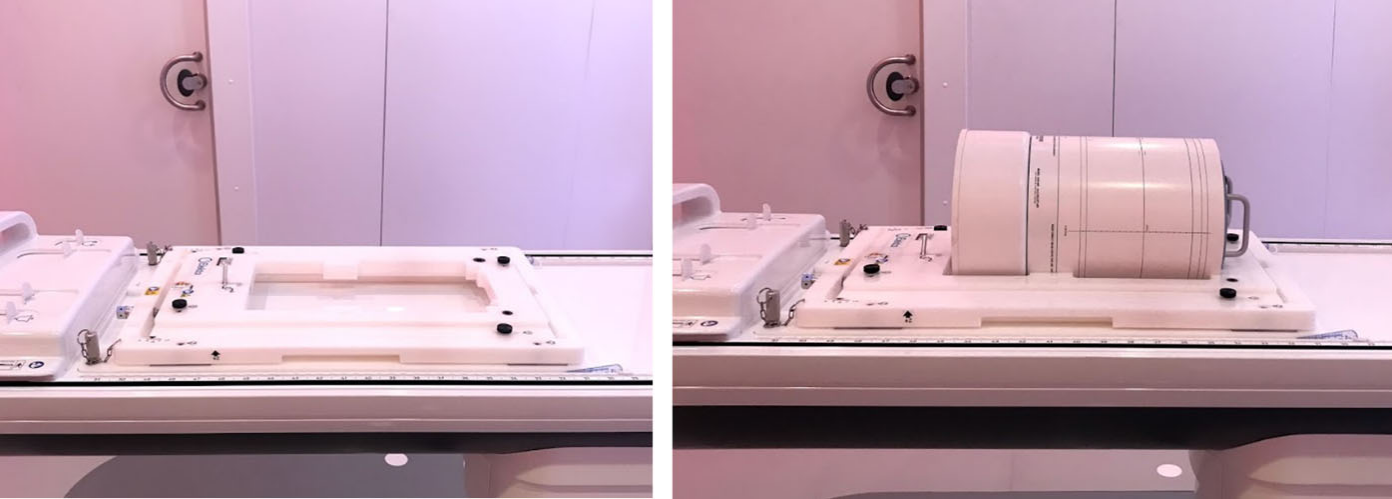
QA platform (left) and QA platform with ArcCheck phantom (right).

Prior to any treatment, the reference treatment plan was recalculated on the MR‐compatible SunNuclear ArcCheck (Melbourne, FL), measured and compared using a gamma criterion of 3%/2 mm with global normalization and a 10% low dose threshold. Due to the design of the Unity MR‐linac, the ArcCheck phantom is first placed on the QA platform and translated into the bore. A CT scan of the ArcCheck was imported into the Monaco treatment planning system (TPS) and the relative electron densities were determined as described by Snyder et al.^14^, ^15^ The alignment of the ArcCheck phantom utilizes a custom QA platform since the Unity MR‐linac only has a sagittal laser, which is insufficient to properly align the phantom by itself. As a result, the alignment reproducibility is dependent on the consistency of how the ArcCheck phantom and platform are setup on the treatment couch. The setup for the ArcCheck phantom for QA measurements is shown in Fig. 1

#### 2.A.2 IMRT QA Plan measurement uncertainty

**Fig. 2:**
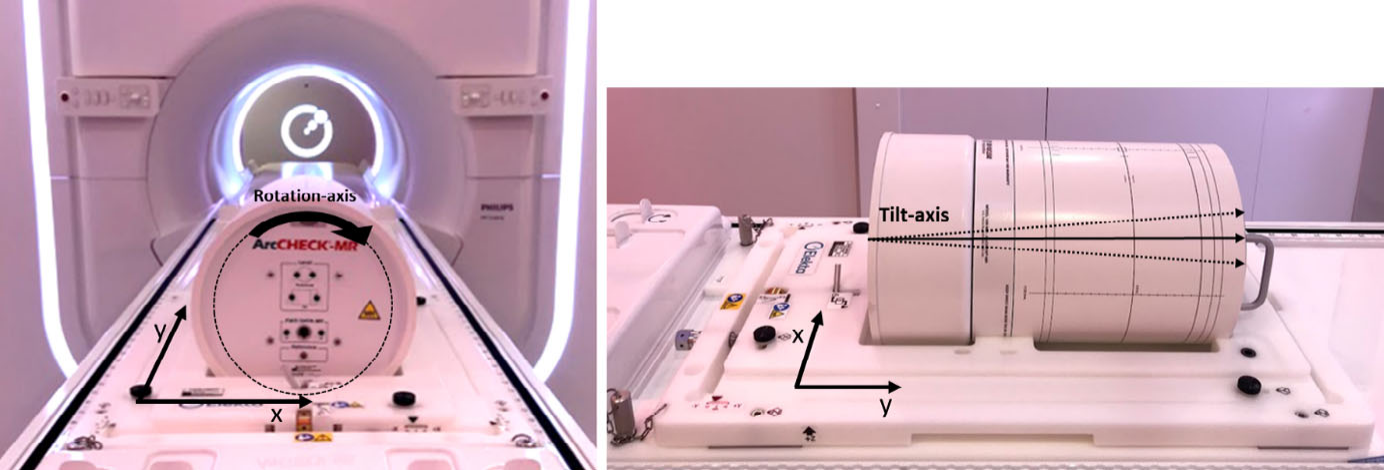
ArcCheck phantom in the QA platform showing the directional components of each device.

The QA platform was calibrated to isocenter during commissioning and verified on a monthly basis to ensure it was within recommended tolerances. The QA platform calibration process consists of placing a phantom supplied by Elekta with radio‐opaque BBs at known locations onto the platform and imaging with the integrated electronic portal imaging device (EPID) panel for various gantry angles. Misalignment in any of the three translational directions of the QA platform from isocenter can be determined by analyzing the projected BB locations at each known gantry angle. Any misalignment is corrected by physically adjusting the QA platform in any of the translational directions and re‐imaging until the alignment is within 0.3 mm in any direction. The ArcCheck rotation and tilt tolerances were calculated by converting the setup tolerances (in degrees) to lengths by projecting onto the flattened SNC analysis plane using the known dimensions of the ArcCheck. This was necessary because the SNC patient software preforms a 2‐dimensional gamma analysis by displaying the cylindrical dose collected and maps it onto a 2‐dimensional plane. The z‐direction was not considered because it is a fixed distance and the ArcCheck software does not provide z‐directional shifts. The QA platform setup tolerances are in Cartesian coordinates since it can only be adjusted in the X, Y, or Z dimensions. Figure 2 shows the directional components of the QA platform and the ArcCheck phantom. Table 1 outlines the tolerances, separated into the directional component, for both the QA platform and the ArcCheck phantom.

**Table 1:** Directional Tolerances for ArcCheck phantom and QA platform.

Directional component	Tolerance	Tolerance in length
ArcCheck rotation	±0.5°	±1.16 mm in X
ArcCheck tilt	±0.25°	±1.93 mm in Y
QA platform translation	±0.3 mm	±0.3 mm in X
QA platform translation	±0.3 mm	±0.3 mm in Y

The total directional (X or Y) uncertainty was calculated using the following equation.

(1) $$\sigma_{\mathrm{total}}=\sqrt{\sigma_{\mathrm{AC}}^{2}+\sigma_{\mathrm{QAP}}^{2}}$$

where $\sigma_{\mathrm{AC}}$ is the directional component uncertainty of the ArcCheck phantom and $\sigma_{\mathrm{QAP}}$ is the directional component uncertainty of the QA platform. Table 2 outlines the total uncertainty ($\sigma_{\mathrm{total}}$) calculated for both the X and Y directions.

**Table 2:** Total uncertainty of the ArcCheck phantom setup for the X and Y directions.

**Total uncertainty**	
x‐direction	1.20 mm
y‐direction	1.95 mm

The SNC software allows for a virtual shift of the measured data in both the X and Y directions.

Based on the above uncertainty analysis, up to a 2 mm shift in either the X or Y directions is allowed.

The gamma pass rates were recorded for all reference and adapted treatment plans. AAPM TG‐218 guidelines were used when analyzing the data. SPC methodology was further used to compare the adaptive plan IMRT QA measurements to the original reference plan measurements. All measurements were performed on the MR‐compatible SunNuclear ArcCheck with a gamma criterion of 3%/2 mm using a global normalization and a 10% low dose threshold.

#### 2.A.3 Statistical process control analysis

Traditionally, SPC utilizes the lower control limit (LCL), upper control limit (UCL), and the center line. The center line was defined to be the mean of the reference data. The LCL was determined using data from the reference IMRT measurements,

(2) $$LCL=\mu_{w}‐L\sigma_{w},$$

where $\mu_{w}$ is the mean of the reference data, $\sigma_{w}$ is the standard deviation of the reference data, and L is the desired distance of control limits from the central line, or in other words the number of standard deviations. The calculation for this study was based on an L of 3 representing three standard deviations from the mean. Because there is no clinical significance of an upper bound when examining gamma pass rates, the UCL was not calculated.

In addition, a one‐sided process capability ratio $\left(C_{p,l}\right)$ was used to assess the adaptive plan results,

(3) $$C_{p,l}=\frac{\mu ‐LCL}{3\sigma },$$

where μ is the mean of the experimental data, σ is the standard deviation of the experimental data, and LCLis the value calculated from the reference data using Eq. (2). A $C_{p,l}$ value above 1 indicates that the variability of the test data, adaptive plan gamma pass rates, was within the inherent variability of the process (reference plan pass rates).

For the SPC analysis, the process capability ratios were determined for ATP and ATS plans using all of the measured data. When calculating the process capability ratios for the SBRT and conventional fractionation plans, only the first fraction of the week was used in the conventional fractionation arm to balance the number of measurements between the shorter fraction SBRT and longer conventional fractionation schemes.

#### 2.A.4 Correlation between QA gamma pass rates and plan complexity

Plan complexity was determined through calculation of the modulation degree, as given in Eq. (4), for each reference, ATP, and ATS plan in this study.

(4) $$modulation\;degree=\frac{MU_{\mathrm{total}}}{\sum_{i}^{\mathrm{beam}}UArea_{i}\sum_{j}^{\mathrm{segment}}Area_{i,j}\times MU_{i,j}}$$

In Eq. (4), $MU_{\mathrm{total}}$ represents the total number of MUs for the plan, $UArea_{i}$ is the open beam aperture area for beam i which is a union of all the segments for that beam, $Area_{i,j}$ is the aperture area for segment j in beam i, and $MU_{i,j}$ is the MU associated with segment j in beam i. The beam‐averaged aperture area was defined as the average of all beam specific aperture areas for a given plan. A Spearman’s rank correlation coefficient was used to determine if there was any correlation between modulation degree or beam‐averaged aperture area to the measured gamma pass rates on the Sun Nuclear ArcCheck.

## 3 RESULTS

### 3.A SPC analysis of Elekta unity adapted plans

**Fig. 3:**
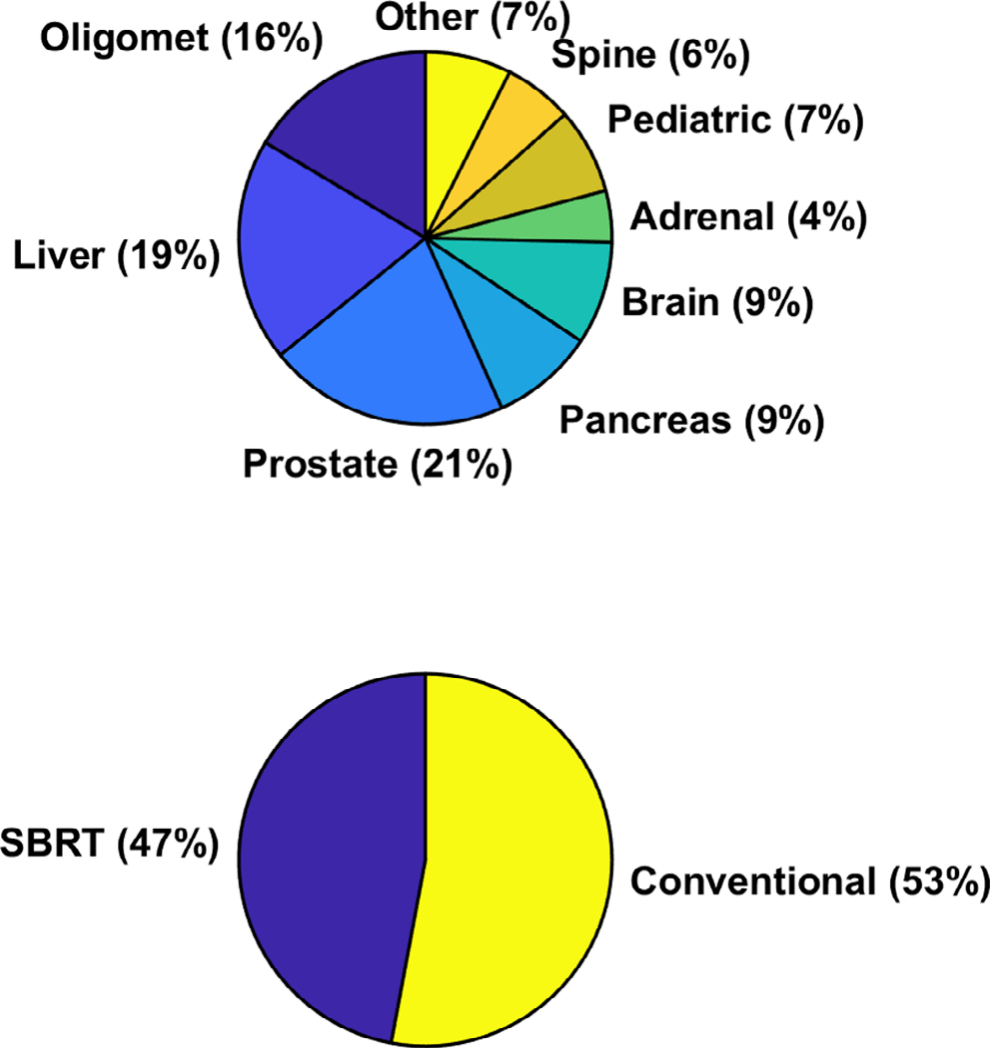
Patient specific treatment sites on the Unity MR‐linac, number of SBRT vs conventional fractionation reference treatment plans.

A total of 65 subjects were a part of this initial MR‐linac study. The patients’ age ranged from 3 to 89 yr with a median age of 67.5 yr. Some patients had boost plans or multiple treatment sites, resulting in a total of 68 reference plans. Any boost or multiple site treatment resulted in a new treatment plan and was thus treated as a new data point. Figure 3 depicts the sites that were treated and the ratio of SBRT to conventional fractionation treatment plans. Of the 68 reference plans, 32 (47%) were SBRT treatments, while the remaining 36 (53%) were standard fractionation schemes.

**Fig. 4:**
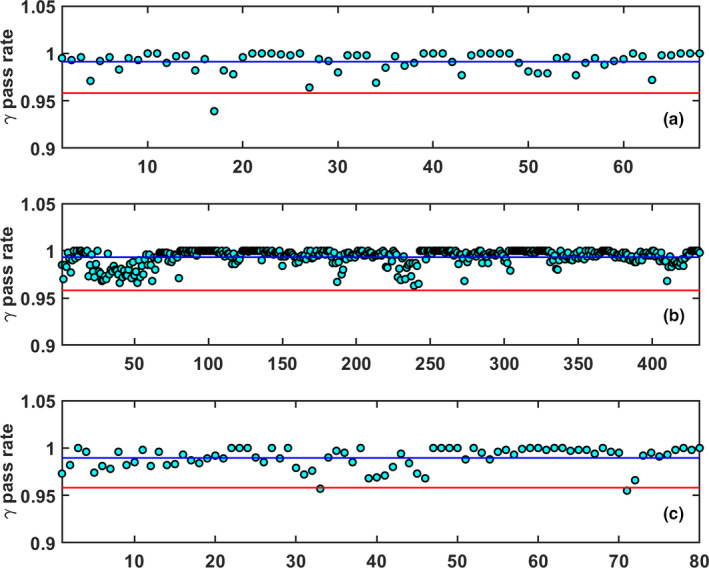
The central line (CL) is represented by the mean of the data (blue), and the LCL is equal to 0.958 (red) (a): 3%/2 mm gamma pass rate for the 68 initial reference treatment plans. (b): 3%/2 mm gamma pass rate for the 432 ATP treatment plans. (c): 3%/2 mm gamma pass rate for the 80 ATS treatment plans. The arbitrary plan number for each measured QA is listed on the x‐axis.

For these 65 patients, a total of 432 ATP (84%) and 80 ATS (16%) plans were generated. The mean pass rate for the reference plans was calculated to be 0.991±0.011, while the mean pass rates for the ATP and ATS plans were calculated to be 0.993±0.008 and 0.990±0.011, respectively. When considering SBRT and conventional fractionations, 88 of the adaptive plans were SBRT while 424 were of a conventional fractionation. The mean pass rate for the SBRT plans was 0.989±0.011. When considering only the first conventional fraction of each week, 103 conventional fractionation plans were used in the analysis with a mean pass rate of 0.993±0.008. If all 424 conventional fractionation measurements were considered, the mean pass rate was 0.994±0.009. The QA gamma pass rate data for the reference, ATP and ATS plans are provided in Fig. 4.

**Fig. 5:**
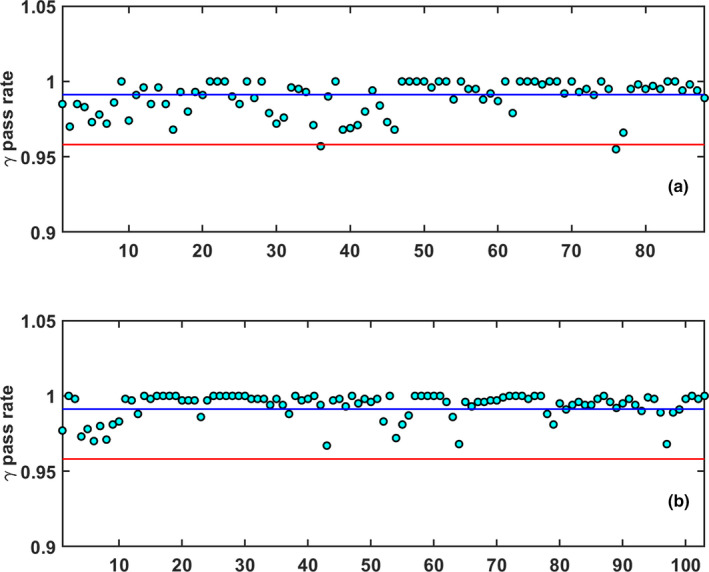
The central line (CL) is represented by the mean of the data (blue), and the LCL is equal to 0.958 (red) (a): 3%/2 mm gamma pass rate for the 88 SBRT treatment plans. (b): 3%/ 2 mm gamma pass rate for the 103 conventional fractionation treatment plans from the first of the week. The arbitrary plan number for each measured QA is listed on the x‐axis.

The $C_{p,l}$, determined for the 432 measured ATP plans and 80 measured ATS plans, was calculated to be 1.403 and 0.940, respectively. The $C_{p,l}$ for the SBRT and conventional fractionation schemes was determined to be 0.902 and 1.383 respectively. Figure 5 shows the gamma pass rates for the SBRT and conventional fractionation analysis.

### 3.B Analysis of plan complexity with QA pass rates

**Fig. 6:**
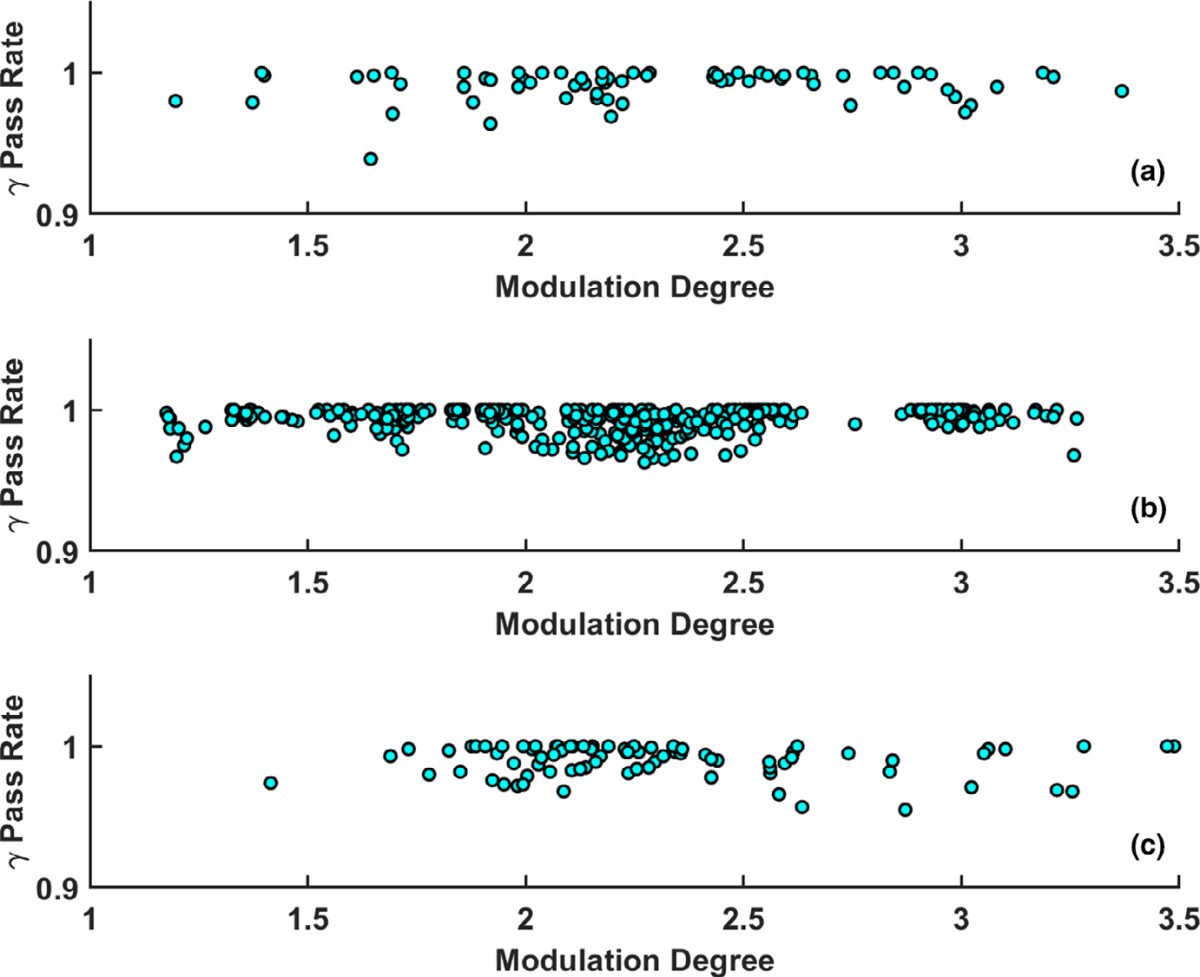
Plots of plan modulation degree against 3%/2 mm gamma pass rates for (a) reference plans, (b) ATP, and (c) ATS plans.

The maximum and minimum IMRT modulation degree was calculated to be 3.49 and 1.18, respectively, where the maximum modulation degree came from a pancreas SBRT plan. The plan modulation degree against gamma pass rate is shown in Fig. 6. A Spearman’s rank correlation was performed to determine if any correlation existed between the plan modulation degree and gamma pass rate. The Spearman’s rank coefficients were calculated to be $0.1\left(p\;=\;0.4\right)$, $0.07\left(p\;=\;0.17\right)$, and $‐0.07\left(p\;=\;0.53\right)$ for the reference, ATP, and ATS plans respectively. The beam‐averaged aperture area for the SBRT and conventional fractionation plans was calculated to be $39.3\pm 24.7\;{\text{cm}}^{2}$ and $66.8\;\pm \;39.0\;{\text{cm}}^{2}$, respectively, and $50.0\;\pm \;33.3{\text{cm}}^{2}$ and $64.3\;\pm \;38.9\;{\text{cm}}^{2}$ for ATS and ATP plans respectively.

**Fig. 7:**
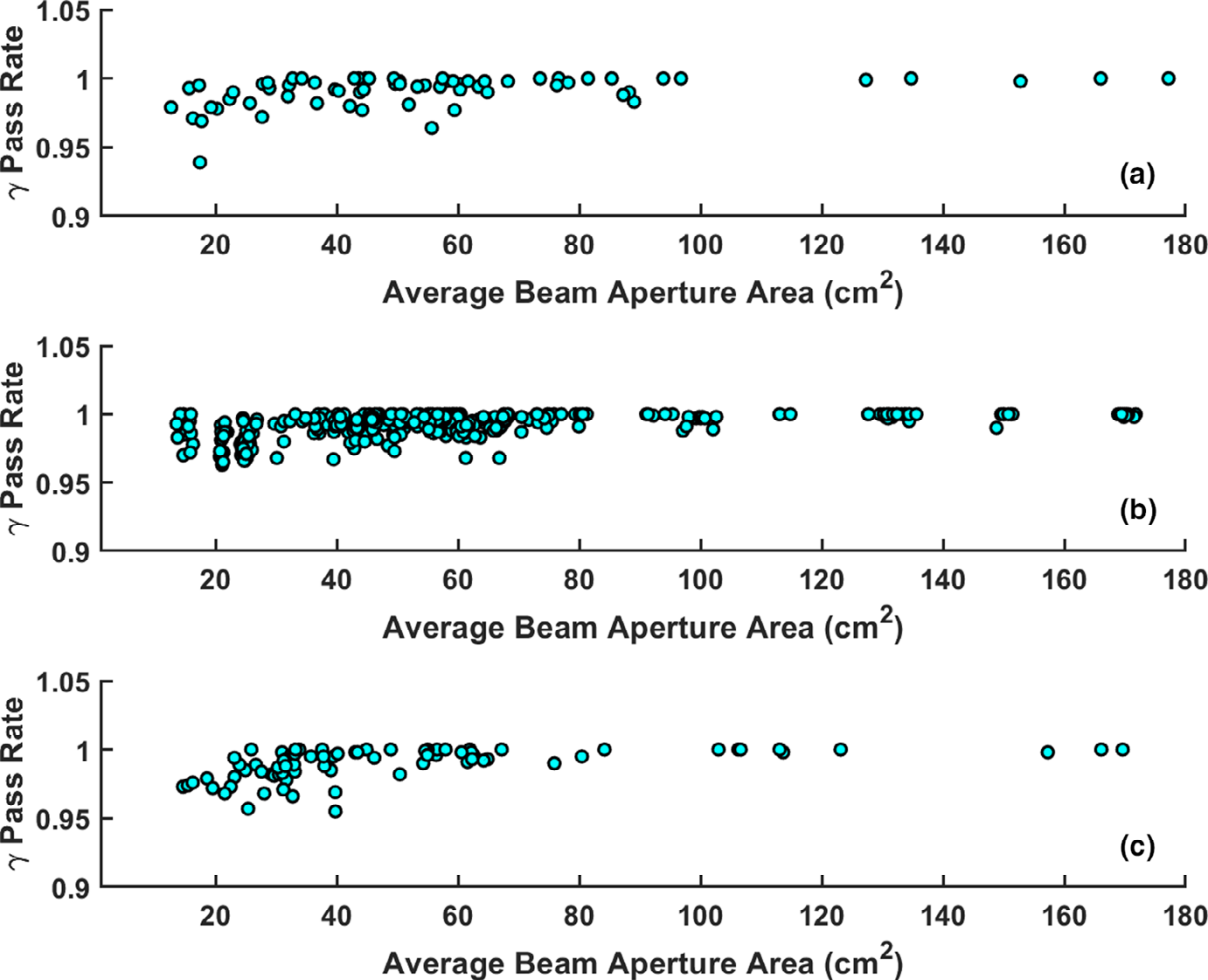
Plots of the average beam aperture area against 3%/2 mm gamma pass rates for (a) reference plans, (b) ATP, and (c) ATS plans.

Analysis of the QA pass rate compared to the beam‐averaged aperture area (Fig. 7) using a Spearman’s rank correlation yielded coefficients of $0.5\left(p\;=\;10^{‐5}\right)$, $0.57(p\;<\;10^{‐6})$, and $0.68(p<10^{‐6})$ for the reference, ATP and ATS plans respectively. Thus, there is a mild, but statistically significant, correlation between gamma pass rate and beam‐averaged aperture area.

### 3.C Sensitivity to machine issues

The initial patient‐specific QA performed for one reference and a few adapted plans showed pass rates below the TG‐218 recommended tolerance of 0.950. Despite these measurements being above the TG‐218 action threshold, they were suspiciously low compared to the other measurements. Upon further investigation, many beam interrupts resulting from gantry encoder errors occurred during the delivery of these QA plans. It was ultimately determined that a loose gantry drive gear was causing the machine faults and the gantry drive assembly was replaced. All plans measured during the time period associated with the loose gantry drive gear were reran with a fully functioning machine and no errors were observed during the delivery of these QA measurements (Table 3). The reran plan gamma pass rates were statistically significantly higher than the initial gamma pass rates ($p=7\times 10^{‐6}$, paired *T*‐test).

**Table 3:** Treatment Fractions initial and remeasured gamma pass rates.

Patient name	Site	Fraction number	Plan Type	Initial pass rate	Remeasured pass rate	Percentage change
Patient 1	Oligomet	0	REF	95.7%	99.3%	+3.60%
		1	ATP	89.4%	98.5%	+9.10%
		2	ATP	93.9%	97.0%	+3.10%
		3	ATP	94.7%	98.5%	+3.80%
		4	ATP	94.2%	98.3%	+4.10%
Patient 2	Brain	3	ATP	92.8%	96.8%	+4.00%
		6	ATP	95.6%	97.0%	+1.40%
		14	ATP	94.5%	97.5%	+3.00%
		16	ATP	94.2%	98.1%	+3.90%
		20	ATP	96.2%	97.4%	+1.20%
		43	ATS	96.3%	98.3%	+2.00%
Patient 3	Prostate	4	ATP	95.3%	99.4%	+4.10%
Patient 4	Prostate	10	ATP	95.4%	99.2%	+3.80%
Patient 5	Oligomet	3	ATP	95.7%	98.6%	+2.90%

## 4 DISCUSSION

SPC methodology is a useful tool to extract details about a process such as patient‐specific QA results. From statistical methods, a lower control limit, which in this study represents three standard deviations from the mean, can be defined. Using the process capability ratio $C_{p,l}$, additional populations can be compared to a reference population such as the reference IMRT QA pass rate. The SPC methodology was useful in this work to highlight differences between the different adaptive planning methods and fractionation schemes. However, it should be noted that based on Eq. (3) that the $C_{p,l}$ is very sensitive to the standard deviation and the degrees of freedom of the sample population in question. Thus, one consideration when using the $C_{p,l}$ metric as an evaluation tool is its sensitivity to minor changes in population statistics.

Based on the results, ATS and SBRT adaptive plans had an increased variability compared to the reference plans which resulted in $C_{p,l}$ values below 1.0. 50% of the ATS adaptive plans (40 of 80) were of SBRT plans with the smaller average beam aperture, whereas only 11% of the ATP adaptive plans (48 out of 432) were SBRT. Thus, it seems as though SBRT plans, with the smaller beam‐averaged aperture area, is driving the $C_{p,l}$ values of the ATS plans below 1.0. Through the Spearman’s rank correlation test, the beam aperture area was mildly correlated with gamma pass rate, where smaller beam aperture areas led to a lower gamma pass rate. These results were found to be statistically significant. It was also found that gamma pass rate was not correlated with modulation factor for the step‐and‐shoot IMRT plans studied. Beam aperture area was observed to have a more significant correlation to the gamma pass rates than the modulation degree. SBRT plans had the lowest beam‐averaged aperture area and the lowest $C_{p,l}$ of 0.902. Similarly, ATS plans had a slightly larger beam‐averaged aperture area and a correspondingly higher $C_{p,l}$ of 0.940.

In the context of this work, the one‐sided process capability ratio can be used to describe how much of a distribution of adaptive plans’ gamma pass rates are contained within the variability of the reference plans’ gamma pass rates. For example, a $C_{p,l}$value equal to 1.00, 0.67, and 0.33 indicate that the gamma pass rates measured for a subset of adaptive plans were all above the LCL, which was set from the distribution of gamma pass rates for the reference plans at the one‐side confidence level of k = 3, k = 2, and k = 1 respectively. Assuming that the distribution of measured gamma pass rates is Gaussian distributed, a $C_{p,l}$that is > 1 would indicate that at least 99.87% of adaptive plans have gamma pass rates above the LCL value of 95.8%. The $C_{p,l}$values of 0.902 and 0.940 from the SBRT and ATS plans, while less than 1, are still strong indicators that these adapted treatment plans will likely have gamma pass rates that are above the LCL set from the reference plans. Specifically, a $C_{p,l}$value of 0.902 and 0.940 corresponds to a one‐sided confidence level of 99.66% and 99.76% respectively. Based on these results, there is a reasonable statistical confidence that a given adaptive treatment plan will provide an acceptable gamma pass rate so long as their reference plan achieves an acceptable gamma pass rate. Thus, SPC is a particularly useful tool that can be used to provide confidence in the deliverability of an adaptive treatment plan, especially given the inability to perform patient‐specific quality assurances measurements prior to each adaptive treatment plan within the MRIgRT workflow.

In connection to the AAPM TG‐218 report, the LCL can be adjusted to reflect either a tolerance or action level. In this context, the value of the $C_{p,l}$ describes the ratio of adapted plans, for a particular subset, that have values greater than the LCL, which can arbitrarily be set. The LCL for the reference plans in this work was calculated to be 0.958, which is well‐above the TG‐218 action threshold. Additionally, all adaptive plan gamma pass rates were above the TG‐218 tolerance level of 0.950. Thus, we can see that the Unity MR‐linac could deliver all adaptive plans generated by the Monaco treatment planning system within clinical standards.

An evaluation of the gamma pass rates for QA plans that were delivered during the period of gantry encoder errors showed suspiciously lower pass rates for a few deliveries. During this time, our standard gantry spoke shot and Winston‐Lutz tests were performed but did not identify any issues with the machine delivery. It is reasonable to think that the loose gantry drive gear caused errors in the gantry position during delivery, but this would only yield slight differences in the spoke shot angles or Winston‐Lutz angles delivered. Consequently, this would be unlikely to cause detectable errors in these standard tests. However, as described here, the patient‐specific QA was able to identify that an error was indeed occurring that was impacting the delivery of treatment plans.

## 5 CONCLUSION

As radiation oncology treatment planning continues to evolve to include MRIgRT patient‐specific treatment plans, clinics not only need to have confidence in the adapted treatment plan, but also in their treatment machine. This paper presents the results from our institution for the first 68 patients on the Unity MR‐linac. The lower control limit (LCL) for the reference plans were determined to be 0.958, and the process capability ratio for ATP and ATS was found to be 1.403 and 0.940 respectively. When analyzing the data as SBRT or conventional fractionation schemes, the $C_{p,l}$ was found to be 0.902 and 1.383, respectively. All measurements were above TG‐218 recommended tolerance providing confidence in the Unity MR‐linac’s performance in generating and delivering adapted plans. It was found that the beam‐averaged aperture area was correlated with QA pass rate, where smaller aperture areas led to lower pass rates. In our analysis, both ATS and SBRT plans measured during this study have lower beam‐averaged aperture areas and $C_{p,l}<1.0$. However, all adaptive plan gamma pass rates were above TG‐218 recommendations and the SPC analysis shows that adaptive plans can be expected to have acceptable pass rates provided the reference plans do. It was also found that modulation degree was not correlated with QA pass rate for either reference or adaptive plans.

## AUTHOR CONTRIBUTIONS

We confirm that all coauthors contributed this work and agreed with the submission of this manuscript to JACMP.

## CONFLICT OF INTEREST

No conflict of interest.
